# Predict New Therapeutic Drugs for Hepatocellular Carcinoma Based on Gene Mutation and Expression

**DOI:** 10.3389/fbioe.2020.00008

**Published:** 2020-01-28

**Authors:** Liang Yu, Fengdan Xu, Lin Gao

**Affiliations:** School of Computer Science and Technology, Xidian University, Xi'an, China

**Keywords:** hepatocellular carcinoma (HCC), drug repositioning, mutated genes, kernel genes, gene expression

## Abstract

Hepatocellular carcinoma (HCC) is the fourth most common primary liver tumor and is an important medical problem worldwide. However, the use of current therapies for HCC is no possible to be cured, and despite numerous attempts and clinical trials, there are not so many approved targeted treatments for HCC. So, it is necessary to identify additional treatment strategies to prevent the growth of HCC tumors. We are looking for a systematic drug repositioning bioinformatics method to identify new drug candidates for the treatment of HCC, which considers not only aberrant genomic information, but also the changes of transcriptional landscapes. First, we screen the collection of HCC feature genes, i.e., kernel genes, which frequently mutated in most samples of HCC based on human mutation data. Then, the gene expression data of HCC in TCGA are combined to classify the kernel genes of HCC. Finally, the therapeutic score (*TS*) of each drug is calculated based on the kolmogorov-smirnov statistical method. Using this strategy, we identify five drugs that associated with HCC, including three drugs that could treat HCC and two drugs that might have side-effect on HCC. In addition, we also make Connectivity Map (CMap) profiles similarity analysis and KEGG enrichment analysis on drug targets. All these findings suggest that our approach is effective for accurate predicting novel therapeutic options for HCC and easily to be extended to other tumors.

## Introduction

Identifying a cure for cancer is a difficult, costly and often inefficient process (Adams and Brantner, 2006). Drug repositioning, i.e., the discovery of new indications of existing drugs, beyond their original indications, is an increasingly attractive new-use discovery model. In addition to saving time and money, one advantage of the drug reuse approach is that existing drugs have been reviewed for safety, dose and toxicity (Ashburn and Thor, 2004; Fathima et al., 2018; Su et al., 2019; Yu et al., 2019). As a result, repurposed drugs usually go into clinical trials faster than newly developed drugs (Yu et al., 2017a, 2018). The rapid development of genomics has resulted in the generation of genomic and transcription group data from disease samples, normal tissue samples, animal models and cell lines. Transcriptomic profiles, such as gene expression data, are most widely used for drug repositioning (Yu et al., 2016). A key data source behind several re-use efforts is the Connectivity Map (CMap) (Lamb et al., 2006), which generated large-scale gene expression profiles in human cancer cell lines treated with different drug compounds under different conditions. The CMap method attempts to provide a more comprehensive view of this transcription data and use them to connect expression profiles across conditions (Lamb et al., 2006). In particular, it suggests that if there is a strong negative correlation between disease characteristics and drug expression characteristics, the drug may have a therapeutic effect on the disease. For example, by systematically comparing the gene expression characteristics of GEO-derived inflammatory bowel disease (IBD) with the gene expression characteristics of a group of 164 drug compounds from CMap, Dudley et al. (2011) predicted several interesting new drug-disease pairs and, in the IBD preclinical model, validated one pair. Yu et al. (2015) proposed a method that discovered the drug-disease association based on protein complexes. In another case, Jahchan et al. (2013) applied a drug repurposing bioinformatics method to identifying antidepressant drugs for the treatment of small cell lung cancer through querying a large compendium of gene expression profiles. Although many machine learning-based methods have been developed by using features (Zhang et al., 2017, 2018a,b, 2019), more and more literature supports the usage of CMap for drug repositioning; despite this, there are still problems. A candidate can often be strengthened using independent disease signatures. But disease signatures are often selected by statistical methods, they are lack of biological information.

Hepatocellular carcinoma (HCC) is the fourth most common primary liver tumor and is an important medical problem worldwide (El-Serag and Mason, 1999; Yu et al., 2017b). HCC is usually caused by infection with hepatitis B virus (HBV) (Chang and Liu, 2016) and hepatitis C (HCV) (Lingala and Ghany, 2015), exposure to aflatoxin B1 from Aspergillus (Kew, 2013), alcohol abuse (Abenavoli et al., 2016), or non-alcoholic fatty hepatitis (Charrez et al., 2016). However, the use of current therapies for HCC is no possible to be cured, and despite numerous attempts and clinical trials, there are not so many approved targeted treatments for HCC. So, it is necessary to identify additional treatment strategies to prevent the growth of HCC tumors.

Many diseases, but especially cancer, are related with abnormal genomes and transcription landscapes (Chakravarthi et al., 2016; Tang et al., 2018). In this study, we seek to use systematic drug repositioning bioinformatics to identify new drug candidates for the treatment of HCC. First, we screen the collection of HCC feature genes that frequently mutated in most samples of HCC based on human mutation data. Then, the gene expression data of HCC in TCGA are combined to classify the gene set of HCC. Finally, the therapeutic score (*TS*) of each drug is calculated based on the kolmogorov-smirnov statistical method. Using this strategy, we identified five drugs that associated with HCC, including three drugs that could cure HCC and two drugs that might have bad effect on HCC. In addition, we also make CMap (Lamb et al., 2006) profiles similarity analysis and KEGG enrichment analysis on drug targets. All these findings suggest that our approach is effective for accurate discovering novel therapeutic options for HCC and easily to be extended to other tumors.

## Materials and Methods

### Datasets

#### HCC Gene Expression Data

The Cancer Genome Atlas (TCGA) (Tomczak et al., 2015) is a comprehensive and coordinated effort to accelerate our understanding of the molecular basis of cancer by applying genomic analysis techniques, including large-scale genome sequencing. TCGA researchers aim to catalog and discover major changes to the cancer-causing genome to create a comprehensive “atlas” of cancer genomes. So far, the project analyzed groups of more than 30 human tumors through large-scale genome sequencing and integrated multidimensional analysis.

We download the gene expression profiles of HCC from TCGA, and there are 423 samples in the data set. The type of a sample is distinguished by the barcode provided by TCGA. If the fourth part of the barcode of one sample is in the range from 01 to 09, the sample is a cancer sample. If the fourth part of the barcode in the range from 10 to 19, the sample is a normal sample. The specific introduction to the barcode can be found in TCGA help file. First, we obtain gene expression matrix data (20,501 × 423), which contains 373 cancer samples, 50 normal samples, and 20,501 genes. Then, we standardize the expression values of all genes as follows:

(1) $$\begin{array}{l} z_{ij}=\frac{g_{ij}-mean\left(g_{i}\right)}{std\left(g_{i}\right)} \end{array}$$

where *g*_*ij*_ represents the expression value of gene *i* in sample *j*, and *mean* (*g*_*i*_) and *std* (*g*_*i*_), respectively represent mean and standard deviation of the expression vector for gene *i* across all samples. Finally, we use Limma (Ritchie et al., 2015) to analyze cancer and normal samples and get the log *FC* value of each gene. The definition of log *FC* is as follows:

(2) $$\begin{array}{l} \log FC_{i}=\log_{2}\left(\frac{\frac{1}{\left|T\right|}\underset{k\in T}{\sum }z_{ik}}{\frac{1}{\left|N\right|}\underset{k\in N}{\sum }{\text{z}}_{ik}}\right) \end{array}$$

where log *FC*_*i*_ is the log *FC* value of gene *i*; *z*_*ik*_ is the normalized expression of gene *i* in sample *k* [see formula (1)]; *T* is the set of cancer samples (|*T*| = 373); *N* is the set of normal samples (|*N*| = 50).

For a gene, if its |log *FC*| ≥ 1 and *p*− > *value* ≤ 0.02, it is a differentially expressed gene. The thresholds of log *FC* and *p*− > *value* refer to Dalman et al. (2012).

#### Gene Expression Data Related to Drugs

The gene expression data related to drugs is downloaded from the CMap (http://www.broadinstitute.org/cmap/) database. It contains 6,100 instances which cover 1,309 drugs. These instances are measured on five types of human cancer cell lines, including the breast cancer epithelial cell lines MCF7 and ssMCF7, the prostate cancer epithelial cell line PC3, the nonepithelial lines HL60 (leukemia) and SKMEL5 (melanoma).

#### SNP Mutation Data of HCC

We download the single nucleotide polymorphism (SNP) gene mutation data of HCC from TCGA database. The SNP mutation data contains 373 cancer patient sample files, and each sample file contains the detailed descriptions of all the mutated genes. Since the mutation frequency of each gene across all samples is different, we select genes with relatively high mutation frequency for further analysis. Here, the mutation frequency is set to be no less than 11 (11 = 373 × 3%), that is a gene mutated in at least three percent of all samples. These genes are defined as frequently mutated genes. Finally, we find 406 frequently mutated genes.

### Methods

#### Defining the Feature Gene Set of HCC

According to the data analysis we have done in section **Datasets**, we can divide the 20,501 genes into three classes based on their mutation frequency and differential expression value. One category is the kernel genes, which mutate frequently. The second category is the secondary genes, which do not mutate frequently but differentially express. The third category is the marginal genes, which neither mutate frequently nor differentially express.

In our work, we take the 406 kernel genes, i.e., frequently mutated gene, as the feature gene set of HCC.

#### Calculating the Therapeutic Scores of Drugs

We select kernel genes as the feature genes of HCC and rank them in descending order based on their differential expressions. For a gene, if its log *FC* value is >0, it is stored in up-regulated gene set. Otherwise, it is stored in down-regulated gene set. Finally, we get two ordered gene lists for HCC: the up-regulated gene list (*G*_*up*_) and the down-regulated gene list (*G*_*down*_).

We get 6,100 gene expression instances covered 1,309 drugs from CMap database. In other words, a drug may correspond to multiple instances. We rank the genes in each instance by their differential expression values between drug-treated and drug-untreated cell lines. In this way, we get 6,100 drug-related gene lists. Therefore, based on kernel genes and 6,100 drug-related gene expression instances, we use a non-parameter, ranking-based pattern matching strategy that was originally introduced by Lamb et al. (2006) to evaluate the relationship between drugs and HCC.

We take each ranked drug expression profile as reference signature and assess their similarity to HCC. We compute a connectivity score separately for the set of up- or down-regulated genes: *ES*_*up*_ or *ES*_*down*_. The computational formulas as follows (Lamb et al., 2006):

(3) $$\begin{array}{lll} a & = & \underset{p=1}{\overset{m}{Max}}\left[\frac{p}{m}-\frac{V\left(p\right)}{n}\right] \end{array}$$

(4) $$\begin{array}{lll} b & = & \underset{p=1}{\overset{m}{Max}}\left[\frac{V\left(p\right)}{n}-\frac{p-1}{m}\right] \end{array}$$

(5) $$\begin{array}{l} ES_{up/down}=\{\begin{array}{c} a_{up/down}(\text{if }{\text{a}}_{up/down}>{\text{b}}_{up/down})\\ -b_{up/down}(\text{if }{\text{a}}_{up/down}<{\text{b}}_{up/down}) \end{array} \end{array}$$

Where *n* represents the total number of genes in the reference drug expression profile; *m* represents the size of *G*_*up*_ or the size of *G*_*down*_; *p* represents the position of the input set (*p* = 1…*m*); *V*(*p*) is the position of the *p*th input gene in the gene list of drug expression profile.

The therapeutic score (*TS*) of a drug is calculated as follows:

(6) $$\begin{array}{l} TS=\frac{1}{k}\overset{k}{\underset{j=1}{\sum }}ES_{up}-ES_{down} \end{array}$$

If the up-regulated genes are near the top (up-regulated) of the rank-ordered drug gene lists and the down-regulated genes are near the bottom (down-regulated) of the rank-ordered drug gene lists, we can get high positive therapeutic scores (*TS*), which indicate the drugs and HCC have similar expression profiles and the drugs might aggravate HCC. On the other hand, if the up-regulated pathway genes are near the bottom of the rank-ordered drug gene lists and the down-regulated pathway genes are near the top of the rank-ordered drug gene lists, we can get negative therapeutic scores (*TS*), which imply the given drugs and HCC have adverse expression profiles and the drugs could be treatment candidates for HCC.

## Results

### Analysis of Disease Characteristics of HCC

**Figure 1:**
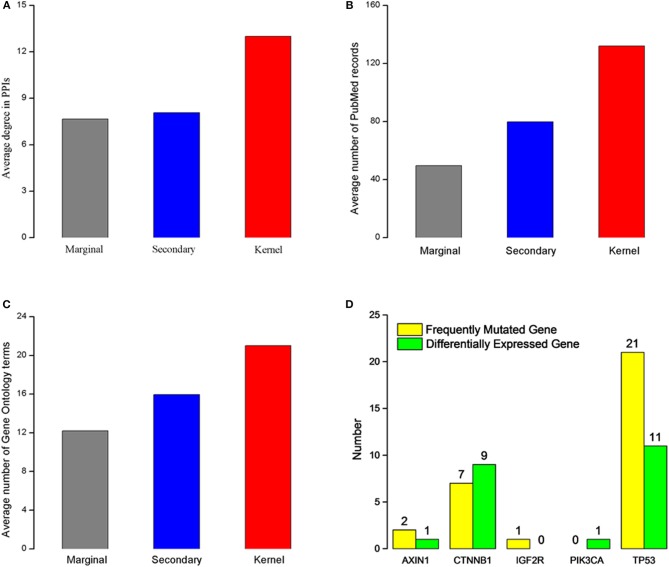
Characteristics of the three gene types. **(A)** Average degree for three different gene types. **(B)** Average PubMed records associated for each gene type. **(C)** Gene Ontology terms annotated for each gene type. **(A–C)** The red rectangle represents kernel genes, the blue rectangle represents secondary genes, the gray rectangle represents marginal genes. **(D)** Type distribution of five kernel genes' direct neighbor genes. Green rectangle represents differentially expressed genes in HCC, and yellow rectangle represents frequently mutated genes in patients with HCC.

We characterize the kernel, secondary, and marginal genes in the context of protein interaction (PPIs) network, PubMed (www.ncbi.nlm.nih.gov/pubmed), and Gene Ontology (Ashburner et al., 2000) term annotation. The Human Protein Reference Database (HPRD) (Prasad et al., 2009) is a protein database for experimentally derived information about human proteomics, including protein and protein interactions (Ding et al., 2016; Wei et al., 2017a), post-translational modifications (PTMs) (Wei et al., 2017b) and other information. We download all human PPIs from this database, containing 15,231 proteins and 38,167 interactions. Interestingly, we find that all three gene types had heterogeneous degree distribution, and that the kernel genes tend to have higher degrees than those of secondary and marginal genes (Figure 1A). Similarly, kernel genes are related with more PubMed records and Gene Ontology term annotation than secondary and marginal genes (Figures 1B,C).

In order to analyze biological functions of kernel genes, we analysis the nine HCC pathogenic genes obtained from Online Mendelian Inheritance in Man (OMIM) (Hamosh et al., 2005) from two aspects of gene mutation and expression level change. These eight HCC pathogenic genes (Table 1) are IGF2R, CASP8, MET, PDGFRL, TP53, PIK3CA, CTNNB1, and AXIN1. We find that five (IGF2R, TP53, PIK3CA, CTNNB1, AXIN1) of these genes are belong to kernel genes, these genes are frequent mutations, but their expression level don't change significantly. For direct neighbors in PPIs of these five genes, we find that there are frequently mutated or differentially expressed genes (see Figure 1D) among their direct neighbors. TP53 is a quite important tumor suppressor gene, which can translate and synthesize protein P53. P53 protein is a vital regulator for cell growth, proliferation and injury repair. For the direct neighbors of TP53, there are 27 frequently mutated genes, and 11 differentially expressed genes. CTNNB1 gene can encode β-catenin, a dual function protein that involves in regulation and coordination of cell–cell adhesion and gene transcription (Nollet et al., 1996). Recent study of HCC has shown that CTNNB1 gene mutations and overexpression of its encoded protein are closely related to occurrence, progression and prognosis of tumor (Kitao et al., 2015). CTNNB1 has 7 frequently mutated direct neighbors, and 9 differentially expressed direct neighbors. The above analysis results show that the kernel genes selected by mutation and expression information contain more comprehensive biological knowledge and to some extent, the characteristics of HCC can be depicted.

**Table 1:** HCC related genes extracted from OMIM.

**Gene names**	**Gene entrez IDs**
IGF2R	3482
CASP8	841
MET	4233
PDGFRL	5157
TP53	7157
PIK3CA	5290
CTNNB1	1499
AXIN1	8312

### Choosing Potential HCC Drugs Through CTD Benchmark

To find most likely HCC-related drugs, we need evaluate the precision of our method firstly. We take Comparative Toxicogenomics Database (CTD) (Davis et al., 2015) as benchmark. CTD supplies manual collated information about drug-gene, drug-disease, and gene-disease interactions. Curated chemical-disease relationships are obtained from the published literature by CTD biocurators and inferred relationships are set up via CTD curated chemical-gene associations.

**Figure 2:**
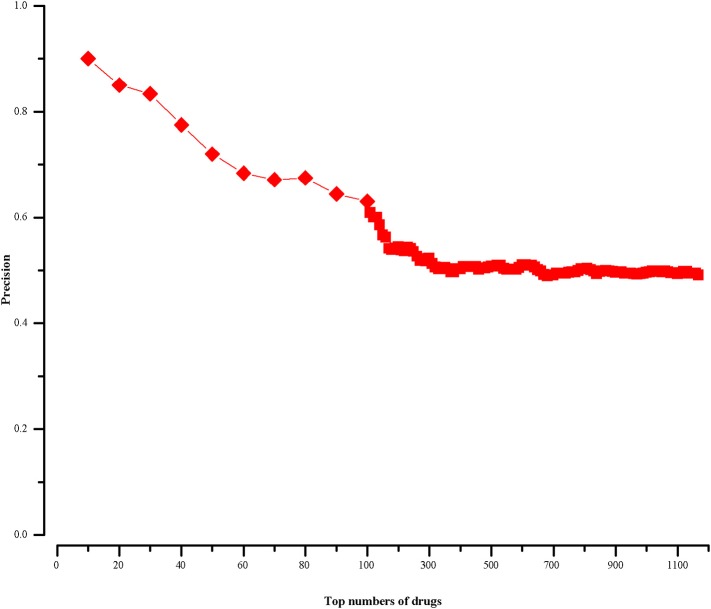
The precision of our approach at different top-x drugs.

For a drug in CMap, if it cannot find corresponding chemical name in CTD, we will not calculate its therapeutic score (defined in section “Methods”). In this way, we finally get 1168 scored drugs. Because most drug-disease associations in CTD are not marked as positive or negative, we rank the 1168 drugs in descending order by the absolute values of their therapeutic scores. We know the top drugs imply stronger connections with HCC. And then we calculate the precisions of our approach at different top-x drugs, which are shown in Figure 2. The precision is calculated as follows:

(7) $$\begin{array}{l} precision=\frac{P_{CTD}}{P} \end{array}$$

where *P* represents the number of top-x drugs, i.e., *P* = x; *P*_*CTD*_ represents the number of drugs in the top-x drugs, which can be found related with HCC in CTD database.

We find in the top-10 drugs (x = 10), there are 9 drugs associated with HCC in CTD. That is to say, the precision is 0.9. For the top-20 drugs (x = 20), the precision is 0.85 and there are three potentially HCC-related drugs. When x is 30, its precision is 0.83 and we get five potential drugs with HCC. From the Figure 2, we notice that with the increase of x, the precision declines and the number of potential drugs increases. We aim to predict relatively more HCC-related drugs with high precision. Then, we choose top-30 (x = 30) drugs for further analysis.

### Validating Potentially HCC-Related Drugs Through Pubmed Literature

In the above section, we choose the top-30 drugs (precision = 0.83) for further analysis. There are 19 therapeutic drugs with negative *TS* values in the top-30 drugs, shown in Table 2. Sixteen of them can be found having connections with HCC in CTD (Davis et al., 2015). Three of the 16 drugs are marked as therapeutic drug (Rank = 1, Rank = 9, Rank = 11, and Evidence = “T” in Table 2) for HCC. Meanwhile, one drug is marked as marker/mechanism drug (Rank = 15, Evidence = “M” in Table 2) for HCC and the other 12 inferred drugs are unmarked in CTD. Here, we can indicate these 12 unmarked drugs are possibly therapeutic drugs for HCC. The rest three drugs (Securinine, Mercaptopurine, and Reserpine) are newly predicted ones by our method, which are marked as bold in Table 2. Based on PubMed, we analyze the three drugs further. PubMed, a free resource, is developed and maintained by the National Center for Biotechnology Information (NCBI) at the National Library of Medicine (NLM). PubMed comprises more than 26 million inferrederences and abstracts on life sciences and biomedical topics.

**Table 2:** Nineteen therapeutic drugs for HCC in the Top-30 drugs.

**Rank**	**Drug name**	**Evidence**	**Inferred count**
1	Daunorubicin	T	42
2	Chrysin	Inferred	34
3	Topiramate	Inferred	8
**4**	**Securinine**	**NULL**	**NULL**
5	Piperlongumine	Inferred	8
6	Luteolin	Inferred	28
7	Apigenin	Inferred	36
8	Celastrol	Inferred	19
9	Sirolimus	T	68
**10**	**Mercaptopurine**	**NULL**	**NULL**
11	Genistein	T	93
12	Irinotecan	Inferred	46
13	Sanguinarine	Inferred	5
14	Tyrphostin Ag-825	Inferred	7
15	Decitabine	M	84
16	Camptothecin	Inferred	28
**17**	**Reserpine**	**NULL**	**NULL**
18	Mycophenolic Acid	Inferred	7
19	Tyrphostin Ag-1478	Inferred	35

*Evidence represents a drug-disease association is curated, inferred or not existed in CTD database. Curated associations include three types: marker/mechanism (Evidence = “M”), therapeutic (Evidence = “T”), marker/mechanism & therapeutic (Evidence = “M&T”). If an association is inferred by CTD, Evidence = “inferred,” and if it is not existed in CTD, Evidence = “NULL”; Inferred Count represents the number of inferrederence (s) for the curated and inferred associations. If an association is not existed in CTD, Inferred Count = “NULL”*.

**Figure 3:**
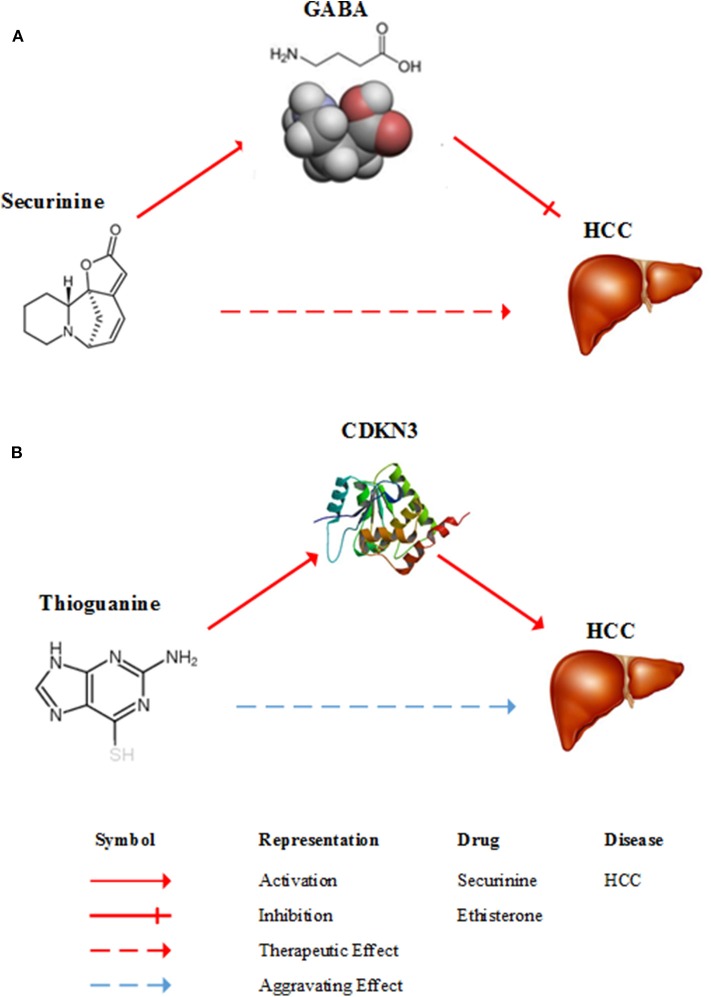
Diagrams of the possible mechanism of between HCC and two drugs. **(A)** A possible mechanism of securinine treating HCC. Securinine has been found to be active as a γ-amino butyric acid (GABA) receptor antagonist. GABA stimulates HCC cell line HepG2 growth. Consequently, it means that securinine is a promising agent with therapeutic effect on HCC patients through inhibiting GABA receptor. **(B)** A possible mechanism of thioguanine aggravating HCC. Thioguanine is a guanine analogs and it can decrease the expression of CDKN3. But, CDKN3 gene inhibits tumor growth by controlling mitosis. Hence, thioguanine may get aggravating effect on HCC patients.

Securinine (Rank = 4 in Table 2), a quinolizine pseudoalkaloid (not from amino acid) from securinega suffurutiosa or securinini nitras, is one of central nervous stimulants and clinically applied to treat amyotrophic lateral sclerosis (ALS) (Buravtseva, 1958), poliomyelitis (Copperman et al., 1973) and multiple sclerosis (Copperman et al., 1974). It is found to be active as a gamma-aminobutyric acid (GABA) receptor antagonist (Perez et al., 2016). GABA is the main inhibitory neurotransmitter of the central nervous system and plays an important role in reducing neuronal excitability throughout the nervous system. Studies show that GABA stimulates HCC cell line HepG2 growth (Lu et al., 2015). Consequently, it means that securinine is a promising agent with therapeutic potential for HCC through inhibiting GABA receptor. Figure 3A gives a diagram of the possible mechanism of the treatment of HCC by securinine.

Mercaptopurine(6-MP, Rank = 10 in Table 2) is a drug for cancer and autoimmune diseases (Sahasranaman et al., 2008). As a purine analog, mercaptopurine belongs to purine antagonist anti-metabolic drugs (Thackery, 2002). 6-MP nucleotides inhibit the synthesis and metabolism of pure nucleotides by inhibiting an enzyme called phosphoribosyl pyrophosphate (PRPP) amidotransferase PRPP Amidotransferase is a rate-limiting enzyme for pure synthesis (Zollner, 1982). This changes the synthesis and function of RNA and DNA. Mercaptopurine interferes with nucleotide conversion and glycoprotein synthesis. This makes the mercaptopurine can effectively inhibit the synthesis of DNA, thereby inhibiting the growth of tumor cells (Cara et al., 2004). At present, although there is no direct experiment that mercaptopurine can inhibit the growth of HCC cells, it is used to treat acute lymphoblastic leukemia (ALL), chronic myeloid leukemia (CML), Crohn's disease and ulcerative colitis (Joint Formulary Committee, 2011). In summary, mercaptopurine is likely to achieve a certain effect on HCC.

Reserpine (Rank = 17 in Table 2) is an antipsychotic and antihypertensive drug (Bridgwater and Sherwood, 1960) used to control hypertension and relieve psychotic symptoms (Arnt et al., 1985). The results of Gwak et al. (2009) showed that reserpine could reduce the expression level of CCND1 gene and its encoded protein. The CCND1 gene encodes the cyclin D1 protein. Cyclin D1 protein is a member of the circulatory protein family, involved in regulating cell cycle progression. This protein plays a key role during the transition from the G1 phase, in which the cell grows, to the S phase, during which DNA is replicated. Overexpression of this protein allows cells to be easily crossed G1/S checkpoint that limits the growth of cells, which promotes tumor hyperplasia and is therefore considered to be an oncoprotein (Donnellan and Chetty, 1998). Some studies have found that CCND1 gene is over-expressed in HCC (Xu et al., 2004). Thus, reserpine can potentially be used as an agent against HCC.

The other 11 drugs with negative *TS* values are shown in Table 3. They are possible to aggravate HCC. Nine of them have been found having relationships with HCC in CTD database and we can infer these relationships are possibly negative. The remaining 2 drugs (Tioguanine, Rifabutin) are newly potential drugs for aggravating HCC marked as bold in Table 3. We will investigate the two drugs (Tioguanine, Rifabutin) based on PubMed.

**Table 3:** Eleven aggravating drugs for HCC in the Top-30 drugs.

**Rank**	**Drug name**	**Evidence**	**Inferred count**
1	Cytochalasin B	Inferred	5
2	Exemestane	Inferred	2
3	Spiperone	Inferred	2
4	Cinchonine	Inferred	1
5	Mepacrine	Inferred	8
**6**	**Tioguanine**	**NULL**	**NULL**
**7**	**Rifabutin**	**NULL**	**NULL**
8	N-Phenylanthranilic Acid	Inferred	1
9	Valinomycin	Inferred	1
10	Betulin	Inferred	2
11	Puromycin	Inferred	13

Tioguanine, also known as thioguanine, (Rank = 6 in Table 3) is a guanine analogs, with cell cycle specificity, for the S cycle of the strongest cell sensitivity. In addition, thioguanine can inhibit the synthesis of guanosine nucleoside, by inhibiting the biological activity of guanylate kinase, the drug can inhibit the guanosine monophosphate (GMP) phosphoric acid to guanosine bisphosphate (GDP) transformation process (Golan, 2011). Thibird is a drug used to treat acute myeloid leukemia (AML) (Gill et al., 1982), acute lymphoblastic leukemia (ALL) (Marmont and Damasio, 1973) and chronic myeloid leukemia (CML) (Yang et al., 2006). In 2005, Ganter et al. showed that CDKN3 expression was significantly decreased after a period of administration of thioguanine (Ganter et al., 2005). The CDKN3 gene inhibits tumor growth by controlling mitosis, which is a tumor suppressor gene (Nalepa et al., 2013). Dai et al. found that CDKN3 expression in patients with HCC was significantly lower than that in normal humans. CDKN3 knockout experiments indicated that CDKN3 could inhibit tumor growth (Dai et al., 2016). A possible mechanism of thioguanine aggravating HCC is shown in Figure 3B. Therefore, in order to ensure the effectiveness of the treatment, clinical patients should avoid HCC patients taking thioguanine.

Rifabutin (Rank = 7 in Table 3) is a piperazine-containing rifamycin derivative, the drug has a broad spectrum of antibacterial activity. It can able to bind to the β-subunit of RNA polymerase and inhibit RNA polymerase activity, thereby reducing the number of RNA synthesis of bacterial (Beard, 2001). Rifabutin has been approved to prevent and treat disseminated infections of mycobacterium mycobacterium complex (MAC) carried by HIV-infected persons (Arevalo et al., 1997), and it is also used to treat multidrug-resistant tuberculosis (Skolik et al., 2005). Kobayashi et al. find that rifabutine will lead to an increase in the expression of cytochrome P450 3A4 (CYP3A4) in liver tissue (Nakajima et al., 2011). CYP3A4 is an important metabolic enzyme, belongs to the cytochrome P450 family. It is also the most important component of adult liver microsomes CYP450, this gene is expressed in the intestinal, liver and kidney (Hashimoto et al., 1993). However, Fanni et al. find a significant increase of expression of CYP3A4 in HCC patients and overexpression of CYP3A4 gene could result in drug degradation or even a decreased therapeutic effect (Fanni et al., 2016). Therefore, for both suffering from HCC and tuberculosis patients, doctors should avoid using rifabutin to treat tuberculosis.

### Analyzing Potentially HCC-Related Drugs Through CMap Database

The CMap database can not only be applied to calculate drug-disease correlations, but also can be used to identify connections between two drugs. In particular, for a same disease, if two drugs have strongly positive relationship, they may have similar effects on this disease. On the contrary, if their relationship is negative, they may have opposite effects. In this section, we further analyze the five predicted drugs (three therapeutic drugs shown in Table 2: securinine, mercaptopurine and reserpine; two aggravating drugs shown in Table 3: tioguanine and rifabutin) based on CMap and estimate their correlations [evaluated by formula (6)] with known HCC drugs marked as “therapeutic” in CTD database. The results are shown in Table 4.

**Table 4:** The relationships of five predicted drugs with known HCC therapeutic drugs in CTD.

**Predicted drugs**	**Known HCC drugs in CTD**	**Connectivity scores**
**Securinine**	Daunorubicin	0.916
	Troglitazone	0.902
	Paclitaxel	0.844
**Mercaptopurine**	Estradiol	0.941
	Dexamethasone	0.926
	Sirolimus	0.845
	Troglitazone	0.833
**Reserpine**	Roxithromycin	0.922
	Resveratrol	0.834
Tioguanine	Genistein	−0.973
	Sirolimus	−0.928
	Indometacin	−0.891
	Paclitaxel	−0.872
Rifabutin	Calcium Folinate	−0.878
	Estradiol	−0.873

*The potentially therapeutic drugs of HCC are marked as bold. The other two drugs are potentially aggravating drugs of HCC*.

For the three potentially therapeutic drugs (securinine, mercaptopurine and reserpine) marked as bold in Table 4, we find that they all have strong positive correlation with known drugs for HCC. Securinine yields highly positive connectivity score [calculated by formula (6)] with drugs daunorubicin, troglitazone and paclitaxel. Mercaptopurine is found having strongly positive relationships with drugs estradiol, dexamethasone, sirolimus, and troglitazone. Reserpine gets high positive connectivity scores with drugs roxithromycin and resveratrol. For the two potentially aggravating drugs (tioguanine and rifabutin) in Table 4, they all have negative relationship with known HCC drugs. Tioguanine has high negative connectivity scores with drugs genistein, sirolimus, indomethacin, and paclitaxel. Rifabutin have clear negative connection scores with drugs calcium folinate and estradiol.

### Overlap Between Pathways Associated With Predicted Drugs and HCC-Related Tissue-Specific Pathways

In this part, we further analyze the relationship between these five drugs (three therapeutic drugs: securinine, mercaptopurine and reserpine; two aggravating drugs: tioguanine and rifabutin) and HCC from the point of view of drug targets. First, we get the target set of drugs from DrugBank (Law et al., 2014) because DrugBank contains the most complete information on drug and drug targets. Then, we use DAVID (Huang et al., 2009) to obtain all the KEGG (Kanehisa et al., 2010) pathways of the drug target. The *p*-value is set to be less than or equal to 0.05. The results are shown in **Table 6**.

From Table 5, it can be seen that securinine and tioguanine have no corresponding target information in the DrugBank database. So we can't enrich their associated pathways. Mercaptopurine has two drug targets, and we find five KEGG pathways related to them. Reserpine has two drug targets, which are included in seven KEGG pathways. Rifabutine has five drug targets, and nine KEGG pathways are enriched to them.

**Table 5:** Pathway enrichment analysis result of five selected drugs.

**Drug name**	**Drug targets**	**KEGG pathways**
Securinine	None	None
Mercaptopurine	HPRT1, PPAT	Purine metabolism; Metabolic pathways; Drug metabolism other enzymes; Alanine aspartate and glutamate metabolism; Biosynthesis of antibiotics
Reserpine	SLC18A2, SLC18A1	Cocaine addiction; Synaptic vesicle cycle; Amphetamine addiction; Serotonergic synapse; Dopaminergic synapse; Parkinson's disease; Alcoholism
Tioguanine	None	None
Rifabutin	rpoA, rpoB, rpoC, HSP90A1, HSP90B1	NOD-like receptor signaling pathway;**Prostate cancer;** Estrogen signaling pathway; Protein processing in endoplasmic reticulum; PI3K-Akt signaling pathway; **Pathways in cancer;** Antigen processing and presentation; Thyroid hormone synthesis; Progesterone-mediated oocyte maturation

*The potentially therapeutic drugs of HCC are marked as bold. The other two drugs are potentially aggravating drugs of HCC. “NULL” represents the drug has no targets in DrugBank at present. Thus, its corresponding KEGG pathway is “NULL” too*.

In order to obtain the tissue-specific KEGG pathways of HCC, firstly, the eight genes (see Table 1) related to HCC are extended through obtaining their direct neighbors in liver-specific protein-protein interaction (PPI) network got from GIANT (Greene et al., 2015). Then, we obtain a subnetwork from the liver PPI network, which contains 57 genes and 838 edges with weight ≥ 0.1. Finally, by using DAVID tool, we obtain 12 KEGG pathways related to the 57 genes (see Table 6). The parameters of DAVID are fixed as: *p*-value = 0.001 and count = 5.

**Table 6:** Twelve enriched tissue-specific KEGG pathways with HCC.

**Pathways**	**Number of HCC-specific genes**	***P*-values**
**Pathways in cancer**	**20**	**3.06E-13**
**Prostate cancer**	**10**	**1.41E-08**
Adherens junction	8	1.44E-06
Endometrial cancer	7	2.15E-06
Colorectal cancer	8	2.62E-06
Apoptosis	8	3.32E-06
Melanoma	7	1.36E-05
Wnt signaling pathway	9	1.41E-05
Cell cycle	7	3.28E-04
Notch signaling pathway	5	4.16E-04
Basal cell carcinoma	5	7.61E-04
Melanogenesis	6	8.61E-04

**Figure 4:**
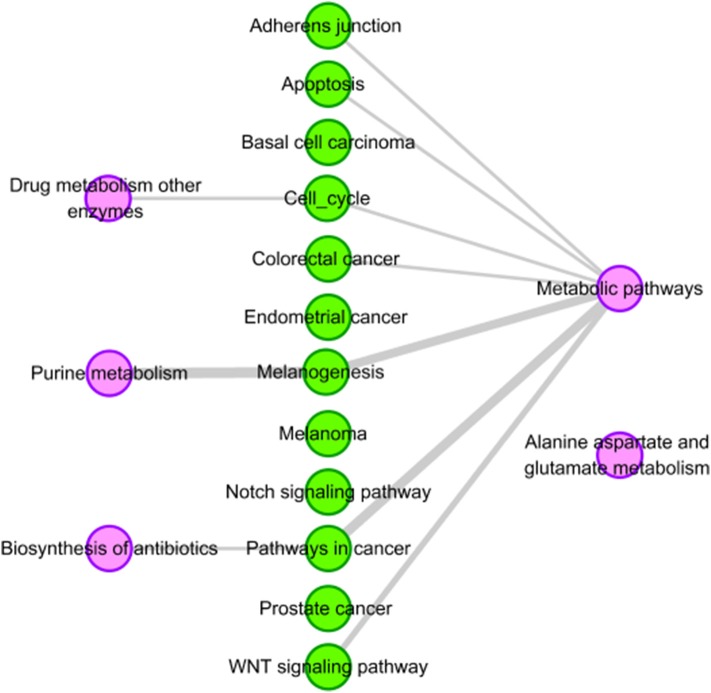
Pathway analysis of mercaptopurine. The purple circles represent pathways related to mercaptopurine, and the green circles represent the tissue-specific KEGG pathways of HCC. The gray edges indicate that there are common genes between two pathways, and the more genes there are, the wider the edges in the network.

We find that there are four pathways related to mercaptopurine have common genes with the 12 tissue-specific KEGG pathway of HCC. The interactions between the four pathways and the 12 tissue-specific KEGG pathway of HCC is shown in Figure 4. The gray edges indicate that there are common genes between two pathways, and the more genes there are, the wider the edges in the network. “Metabolic pathways” have common genes with seven tissue-specific KEGG pathways of HCC. Though there is only one edge between “purine metabolism” and HCC related pathway, the edge is very wide, indicating that there are a lot of common genes. These overlap genes between the pathways of mercaptopurine and HCC tissue-specific KEGG pathways show that mercaptopurine has a potential effect on treating HCC.

**Figure 5:**
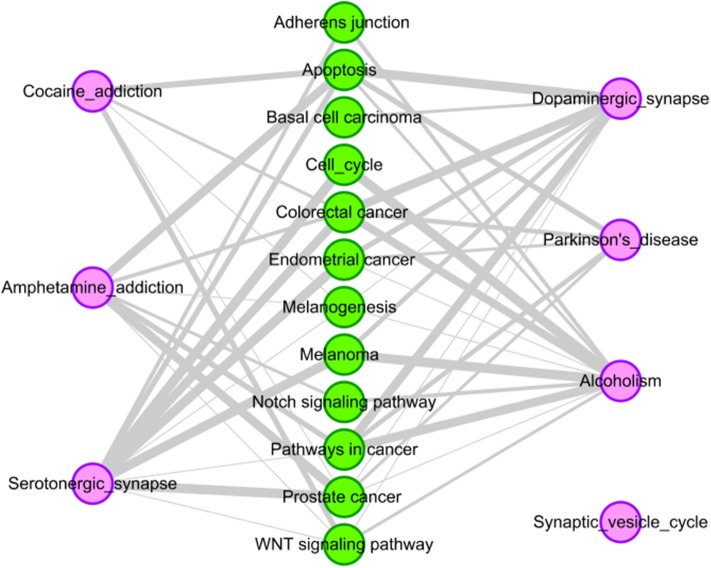
Pathway analysis of reserpine. The purple circles represent pathways of reserpine, and the green circles represent the tissue-specific KEGG pathways of HCC. The gray edges indicate that there are common genes between two pathways, and the more genes there are, the wider the edges in the network.

For drug reserpine, there are six pathways have common genes with the 12 tissue-specific KEGG pathway of HCC. Their relationships are shown Figure 5. For example, “serotonergic synapse” has common genes with ten pathways of HCC. “Dopaminergic synapse” has common genes with nine pathways of HCC. Overall, drug reserpine has more overlapping pathways with HCC, and more genes overlap between pathways. The results indicate that drug reserpine is likely to become the treatment of HCC.

**Figure 6:**
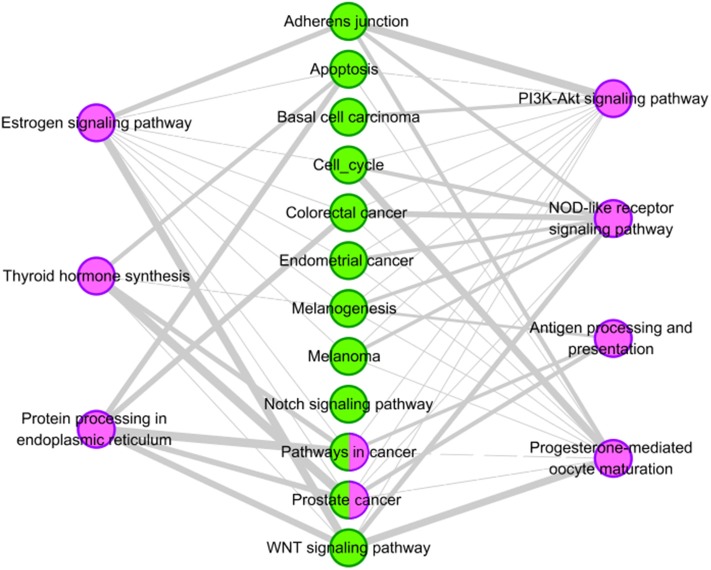
Pathway analysis of rifabutine. The purple circles represent pathways of the rifabutine, and the green circles represent tissue-specific KEGG pathways of HCC. Nodes with two colors represent overlapping pathways for rifabutine and HCC. The gray edges indicate that there are common genes between two pathways, and the more genes there are, the wider the edges in the network.

For the potential aggravating drug rifabutine, we also analyze its pathway overlap with HCC. We try to explain the possible reasons for its aggravating HCC in terms of pathway overlap. Two pathways of rifabutine (“Pathways in cancer” and “Prostate cancer”) are overlapped with pathways of HCC highlighted in Table 6. The interactions between the pathways and the 12 tissue-specific pathways of HCC is shown in Figure 6. Two overlapping pathway nodes are colored in two colors (purple and green) in Figure 6. We find the pathways of rifabutine have a very large number of overlapping genes with the pathways of HCC. This shows a strong correlation between rifabutine and HCC, confirming our prediction on the other hand.

## Discussions

We propose a method based on the combination of gene mutation data and differential expression data. First, we select the feature genes of hepatocellular carcinoma (HCC) that frequently mutated in most samples of HCC based on human somatic mutation data. Then, the gene expression data of HCC in TCGA are combined to classify the genes related to HCC. Finally, the therapeutic score (*TS*) of each drug is calculated based on the kolmogorov-smirnov statistical method. By this method, five drugs associated with HCC are obtained, including three drugs that could be the potential treatment for HCC and two drugs that might have side effect on HCC. There are advantages in our method. First, we take into account the essential impact of genetic changes on HCC. Secondly, we integrate multiple data to define the type of a gene. Finally, our method can clearly distinguish positive and negative relationships between drugs and HCC.

In the future, as more and more drug-related data continues to be generated, such as cell lines, gene expression and mutation data, we will further improve our computational model and predict more accurate potential drugs for the treatment of HCC.

## Data Availability Statement

The raw data supporting the conclusions of this article will be made available by the authors, without undue reservation, to any qualified researcher.

## Author Contributions

LY designed the study. All authors analyzed the data, interpreted the results, wrote the manuscript, read and approved the final manuscript.

### Conflict of Interest

The authors declare that the research was conducted in the absence of any commercial or financial relationships that could be construed as a potential conflict of interest.
